# College and Hospital Notes

**Published:** 1909-10

**Authors:** 


### COLLEGE AND HOSPITAL NOTES.

Dr. D. A. Carmichael, Surgeon Public Health and Marine Hospital Service, announces that the new United States marine hospital at No. 2183 Main street is open for the reception of patients. Patients who desire admission should apply at the office of the service, No. 228 Federal building, between the hours of 9 a. m., and 4 p. m., and on Saturdays between 9 a. m. and 1 p. m. After office hours and on Sundays, applications should be made at the hospital.

The College of the City of New York, St. Nicholas avenue and 139th street, is contributing to the Hudson-Fulton Exhibit. During the Hudson-Fulton celebration and for some weeks thereafter, the College of the City of New York will have on exhibition in its historical museum a collection of charts, views, manuscripts and relics representing old New York. Among the charts will be original prints of New Netherlands and New Amsterdam by Nicholas J. Vischer, about 1650; N. Vischer, 1690; Lotter's "New Jorck," 1720; contemporary plans and views of the revolutionary period showing the movements of Washington and Howe in that vicinity during the campaign of 1776; revolutionary battle relics; portraits, residences and letters of old New Yorkers; bronze busts of Washington, Lincoln and Fulton by Houdon and Volk; and other material suggested by the celebration. Take Sixth avenue Elevated Railway to 140th street, or Broadway subway to One Hundred and Thirty-seventh street.

The New Maternity Department at the Buffalo General Hospital was recently opened for the reception of patients. While the work of preparation was under way several nurses were sent to New York for a course of study in obstetric nursing at the Sloane Maternity Hospital. These nurses have returned crowned with experience of value, including the care of newborn infants.

The wards, as well as the private rooms, are equipped with every faculty for the practice of modern obstetrics. The maternity division is not merely a part of the hospital; it is a department distinct and apart from the medical and surgical divisions of the hospital and remote from them.

